# Baseline characteristics and treatment response predictive of nAMD outcomes with ranibizumab therapy in treatment-naive patients: the RACER subgroup analysis

**DOI:** 10.1186/s12886-023-02780-0

**Published:** 2023-01-27

**Authors:** Ching-Yao Tsai, Chien-Liang Wu, Cheng-Kuo Cheng, Yun-Dun Shen, Wen-Chuan Wu, Pei-Chang Wu, Arslan Tsai, Jiann-Torng Chen

**Affiliations:** 1 Taipei City Hospital, Taipei City, Taiwan; 2 Institute of Public Health, National Yang Ming Chiao Tung University, Taipei City, Taiwan; 3 Department of Business Administration, Fu Jen Catholic University, New Taipei City, Taiwan; 4 Municipal Wan Fang Hospital, Taipei City, Taiwan; 5 Shin Kong Wu Ho-Su Memorial Hospital, Taipei City, Taiwan; 6 Taipei Medical University-Shung Ho Hospital, New Taipei City, Taiwan; 7 Kang-Ning General Hospital, Taipei City, Taiwan; 8 Kaohsiung Medical University Chung-Ho Memorial Hospital, Kaohsiung City, Taiwan; 9 Park One International Hospital, Kaohsiung City, Taiwan; 10 Kaohsiung Chang Gung Memorial Hospital, Kaohsiung City, Taiwan; 11 Clinical Development and Medical Affairs, Novartis Taiwan, Taipei City, Taiwan; 12 Tri-Service General Hospital, Chenggong Road, Taipei City 114, Taiwan; 13 National Defense Medical Center, Taipei City, Taiwan

**Keywords:** Anti-VEGF, BCVA, Clinical practice, CRT, nAMD, RACER, Ranibizumab, Real-world, Taiwan, Treatment switch, Treatment response

## Abstract

**Background:**

The Ranibizumab AMD Clinical Efficacy Study (RACER) conducted in treatment-naive adult Taiwanese patients with neovascular age-related macular degeneration (nAMD) suggested the importance of early and intensive dosing of ranibizumab for optimal treatment outcomes. This subgroup analysis aims to provide clinical information on treatment response that can potentially guide on maintaining the treatment or switching anti-VEGF agents in the real-world setting.

**Methods:**

Visual acuity (VA) and central retinal thickness (CRT) were assessed in the RACER subgroup population. Subgroup analysis sets were categorised based on: (1) baseline best-corrected VA (BCVA; ≤ 48 and > 48 letters); (2) baseline CRT (≤ 325 or > 325 μm); and (3) treatment response after three monthly initial injections: < or ≥ 5-letter gain in BCVA and reduction of < or ≥ 50 μm in CRT.

**Results:**

Patient age, sex, nAMD duration and number of ranibizumab injections did not differ significantly between the treatment subgroups. Poor baseline BCVA (≤ 48 letters) and baseline CRT severity (> 325 µm) were predictors of maximum BCVA gains (9.6 ± 12.9 letters [95%CI: 6.3 to 12.9] and 5.1 ± 18.3 letters [95%CI: − 0.5 to 10.8] at Months 3 and 12, respectively) and better CRT reductions (− 127.6 ± 104.2 µm and − 104.2 ± 107.4 µm at Months 3 and 12, respectively; both *P* < 0.001). For the subgroup showing favourable treatment improvement with BCVA gains ≥ 5 letters after three monthly initial injections, 75.6% of patients maintained follow-up at Month 12 with a mean of 6.5 ± 14.3 letter gains (95% CI: 1.2 to 11.7). The BCVA gains < 5-letter subgroup nevertheless had stable BCVA (0.4 ± 12.1 letter gains) and CRT (− 41.9 ± 61.2 µm) at Month 12, respectively. In the subgroup with ≥ 50 µm CRT reduction after three monthly initial injections, there are significantly higher BCVA improvements vs. the < 50 µm CRT reduction subgroup at Month 3 (5.0 ± 8.6 letter gains vs. 1.5 ± 11.6 letter gains, respectively; intergroup *P* = 0.005).

**Conclusion:**

Lower baseline BCVA and higher baseline CRT were associated with BCVA gains and CRT reductions throughout the 12-month study period. Early CRT improvements after three monthly initial injections were associated with BCVA gains as early as Month 3.

**Supplementary Information:**

The online version contains supplementary material available at 10.1186/s12886-023-02780-0.

## Background

Age-related macular degeneration (AMD) is a chronic progressive disease with a reported annual incidence of 1.59 and 0.23 per 100 person-years for early and late AMD, respectively [1]. Late AMD mainly affects the elderly and progresses to neovascular AMD (nAMD) or wet AMD [2]. nAMD is the leading cause of irreversible vision loss affecting 0.46% − 1.81% of the global population [3]. Late AMD affects approximately 1.9% − 7.3% of individuals over the age of 65 years in Taiwan [4], and with its increase in the aging population projected to surpass the global numbers (36.7% vs. 12%) by 2050, nAMD management becomes critical [5].

Intravitreal injections of anti-vascular endothelial growth factor (anti-VEGF) agents, such as ranibizumab and aflibercept, have been the cornerstone of first-line therapies to suppress or halt disease progression in nAMD [6–8]. Considering the significant disease burden, in 2011 and 2014, the Taiwan government reimbursed ranibizumab and aflibercept, respectively, in the National Health Insurance (NHI) program for nAMD treatment [9]. This program currently covers more than 99% of residents and health care utilities in Taiwan [9, 10]. However, as a limited number of doses are allowed for reimbursement per patient life, the clinical options of switching between the drugs become a pressing issue, as it is not available currently in Taiwan [11].

Treatment of nAMD with anti-VEGF therapies requires multiple injections and can be long-term. Despite the standardised anti-VEGF regimen, studies have shown recurrent fluid exudation and gradual loss of efficacy in a proportion of eyes [11]. Frequent dosing [12] and switching to other anti-VEGFs, especially for the non-responsive eyes, have shown benefits resulting in better treatment outcomes [13, 14].

The Ranibizumab AMD Clinical Efficacy in Real-world practice (RACER) study was designed to evaluate the real-world effectiveness and safety of ranibizumab over 12 months in treatment-naive patients with nAMD who were eligible for the NHI 3 + 4 reimbursement scheme. The study primarily showed that early treatment and frequent dosing can lead to better outcomes [15]. Herein we present the subgroup analysis of the RACER study population, analysing the nAMD treatment outcomes at 3 and 12 months based on baseline best-corrected visual acuity (BCVA), central retinal thickness (CRT), and treatment response.

## Materials and methods

### Study design and reimbursement criteria

RACER was an observational study conducted between May 2014 and May 2017 at seven centres in Taiwan. Eligible patients with nAMD were treated with ranibizumab as per the approved labelling dosage, and eligibility criteria for treatment reimbursement are described in the RACER primary study [15]. NHI reimbursement policy did not allow a switch in anti-VEGF therapy after treatment initiation. The study protocol and amendments were approved by the independent ethics committee (IEC) or institutional review board (IRB) for each site.

### Study population

Treatment-naive adult Taiwanese patients recently diagnosed with visual impairment attributable to nAMD (and no other causes), for whom intravitreal treatment with ranibizumab 0.5 mg was prescribed during routine medical practice, were included in the study. According to the local labeling described in Wu WC et al., 2020 [15], ranibizumab is administered once a month for 3 consecutive months and the patients should be followed up regularly on their visual acuity and disease reactivation, thereafter, with a predominantly PRN retreatment regimen. Previously treated patients or those with concomitant conditions in the study eye that would interfere with treatment outcomes, patients with reported allergies/hypersensitivity to the study drug, and pregnant or lactating women were all excluded from the study [15].

Study analysis sets included: (1) *intent-to-treat (ITT)* population − all patients who received at least one dose of observational drug (anti-VEGF) and had at least one post-baseline assessment of study variables, and (2) *3M3D* population − a subset of the ITT population who received all three doses of anti-VEGF within 3 months without protocol deviation.

For the subgroup analyses, patients were categorised based on (1) baseline BCVA in the ITT population (≤ 48 and > 48 letters); (2) baseline CRT in the ITT population (≤ 325 or > 325 μm); and (3) treatment response in the 3M3D population: < or ≥ 5 letter gain in BCVA (BCVA < 5 letters or BCVA ≥ 5 letters) after the three injections, and reduction < or ≥ 50 μm in CRT (CRT < 50 μm or CRT ≥ 50 μm) after the three injections.

### Study endpoints and assessments

The objective of the RACER subgroup analysis was to evaluate the secondary effectiveness endpoints (mean change from baseline in BCVA and CRT at 3 and 12 months) of ranibizumab 0.5 mg treatment in patients with nAMD. As described above, endpoints were assessed based on the patients’ (1) baseline BCVA; (2) baseline severity of CRT and (3) treatment response. Treatment response was defined as a gain of ≥ 5 letters and ≥ 50 µm CRT reduction after three injections in 3 months. BCVA was assessed using the ETDRS score chart at a testing distance of 4 m. Retinal thickness was assessed using the optical coherence tomography (OCT) and characterization of the lesion was assessed using color fundus photography and fluorescein angiography. Protocols for OCT, colour fundus, and fluorescein angiography were described in detail in the study by Wu WC et al., 2020 [15]. Adverse events (AEs) and serious AEs (SAEs) over the 12-month observational period were monitored.

### Statistical analyses

A sample size of 160 patients was predicted to achieve 80% power to test superiority with a 20% dropout rate. For continuous variables, descriptive statistics including number of observations, mean, median, standard deviation, minimum, maximum and 95% confidence intervals were presented; for categorical variables, count and percentages were used to summarise descriptively. Detailed statistical methods are provided in the Statistical Analysis Plan. The mean dosing and the time to the first retreatment were summarised descriptively. In addition, the efficacies of BCVA at Month 12 and CRT at Months 3 and 12 were evaluated after the initial treatment with ranibizumab. Each efficacy endpoint was analysed by duration and severity of nAMD subgroup at baseline. Criteria for sample size, power calculations and details of statistical analysis have been previously described [15].

## Results

### Patient disposition

A total of 161 patients with signed informed consent were enrolled in the study. The safety analysis set comprised all the enrolled patients who received at least one dose of anti-VEGF (ranibizumab) and had at least one post-baseline safety assessment (*N* = 161, 100%). The ITT population (*n* = 152, 94.4%) consisted of all patients who received at least one dose of ranibizumab and at least one post-baseline assessment of effectiveness variables, while the 3M3D population (*n* = 118 patients; 73.3%) comprised a subset of ITT who received three anti-VEGF injections within the first 3 months without any protocol deviations.

Overall, patient baseline demographics and ocular and disease characteristics have been published previously [15].

### Subgroup analysis by baseline characteristics

#### By baseline BCVA

Patients were categorised into two subgroups based on their VA at baseline: (1) BCVA ≤ 48 letters and (2) BCVA > 48 letters. The patient subgroup with baseline BCVA > 48 letters was significantly younger than those with baseline BCVA ≤ 48 (intergroup *P* = 0.014) (Table 1) for the ITT population. There was no significant difference in the total number of ranibizumab injections between the subgroups intergroup (*P* = 0.985) (Table 1).

**Table 1:** Patient characteristics for subgroups by baseline BCVA (ITT^a^), baseline CRT (ITT^a^) and treatment response in BCVA and CRT at Month 3 (3M3D^b^)

Characteristics	Patient sub-groups		
**ITT**^a^			
	**BCVA ≤ 48 letters,** ***N* = 72**	**BCVA > 48 letters,** ***N* = 80**	***P* value**^†^
Age (years), mean ± SD	74.0 ± 9.2	69.6 ± 11.7	0.014*
Male, n (%)	44 (61.1)	54 (67.5)	0.411
nAMD duration (months), mean ± SD	6.6 ± 21.3	4.6 ± 12.7	0.832
No. of ranibizumab injections, mean ± SD	4.3 ± 1.6	4.4 ± 1.8	0.985
	**CRT ≤ 325 µm,** ***N*= 61**	**CRT > 325 µm,** ***N*= 91**	***P* value**^†^
Age (years), mean ± SD	72.4 ± 11.9	71.1 ± 10.0	0.430
Male, n (%)	44 (72.1)	54 (59.3)	0.106
nAMD duration (months), mean ± SD	5.4 ± 11.9	5.7 ± 20.2	0.015*
No. of ranibizumab injections, mean ± SD	4.5 ± 1.8	4.3 ± 1.6	0.463
**3M3D**^b^			
	**Gain < 5 letters,** ***N* = 65**	**Gain ≥ 5 letters,** ***N* = 41**	***P* value**^†^
Age (years), mean ± SD	71.9 ± 11.2	69.4 ± 11.2	0.271
Male, n (%)	41 (63.1)	31 (75.6)	0.085
Baseline BCVA (letters), mean ± SD	50.7 ± 21.3	45.7 ± 18.2	0.081
Baseline CRT (µm), mean ± SD	360.1 ± 115.9	402.8 ± 151.7^c^	0.067
nAMD duration (months), mean ± SD	7.6 ± 23.2	4.4 ± 13.4	0.335
No. of ranibizumab injections, mean ± SD	4.8 ± 1.6	4.7 ± 1.6	0.662
	**Reduce < 50 µm,** ***N* = 34**	**Reduce ≥ 50 µm,** ***N* = 65**	***P* value**^†^
Age (years), mean ± SD	70.3 ± 9.6	72.1 ± 11.8	0.461
Male, n (%)	22 (64.7)	49 (75.4)	0.263
Baseline BCVA (letters), mean ± SD	51.1 ± 23.2	49.9 ± 17.6^d^	0.781
Baseline CRT (µm), mean ± SD	315.8 ± 76.2	400.4 ± 119.7	< 0.001*
nAMD duration (months), mean ± SD	6.9 ± 14.6	6.4 ± 23.1	0.022*
No. of ranibizumab injections, mean ± SD	5.1 ± 1.6	4.8 ± 1.6	0.857

*BCVA* best-corrected visual acuity, *CRT* central retinal thickness, *ITT* intent-to-treat, *N* total number of patients, *n* number of patients, *nAMD* neovascular age-related macular degeneration, *SD* standard deviation, *VEGF* vascular endothelial growth factors

^†^The difference of continuous variables between treatment groups was compared by independent *t* test at a statistical significance level of 0.05. If the data had not been well modelled by a normal distribution, the Mann–Whitney *U* test would be used

^*^Statistically significant

^a^ITT population is defined as all patients who received at least one dose of observational drug (anti-VEGF) and had at least one post-baseline assessment of the study variables

^b^3M3D population is defined as a subset of ITT population that received all three doses of anti-VEGF within 3 months without protocol deviation

^c^Baseline CRT values among patients with ≥ 5 letters BCVA gain were available only for 40 patients

^d^Baseline BCVA values among patients with ≥ 50 µm CRT reduction were available only for 64 patients

**Fig. 1:**
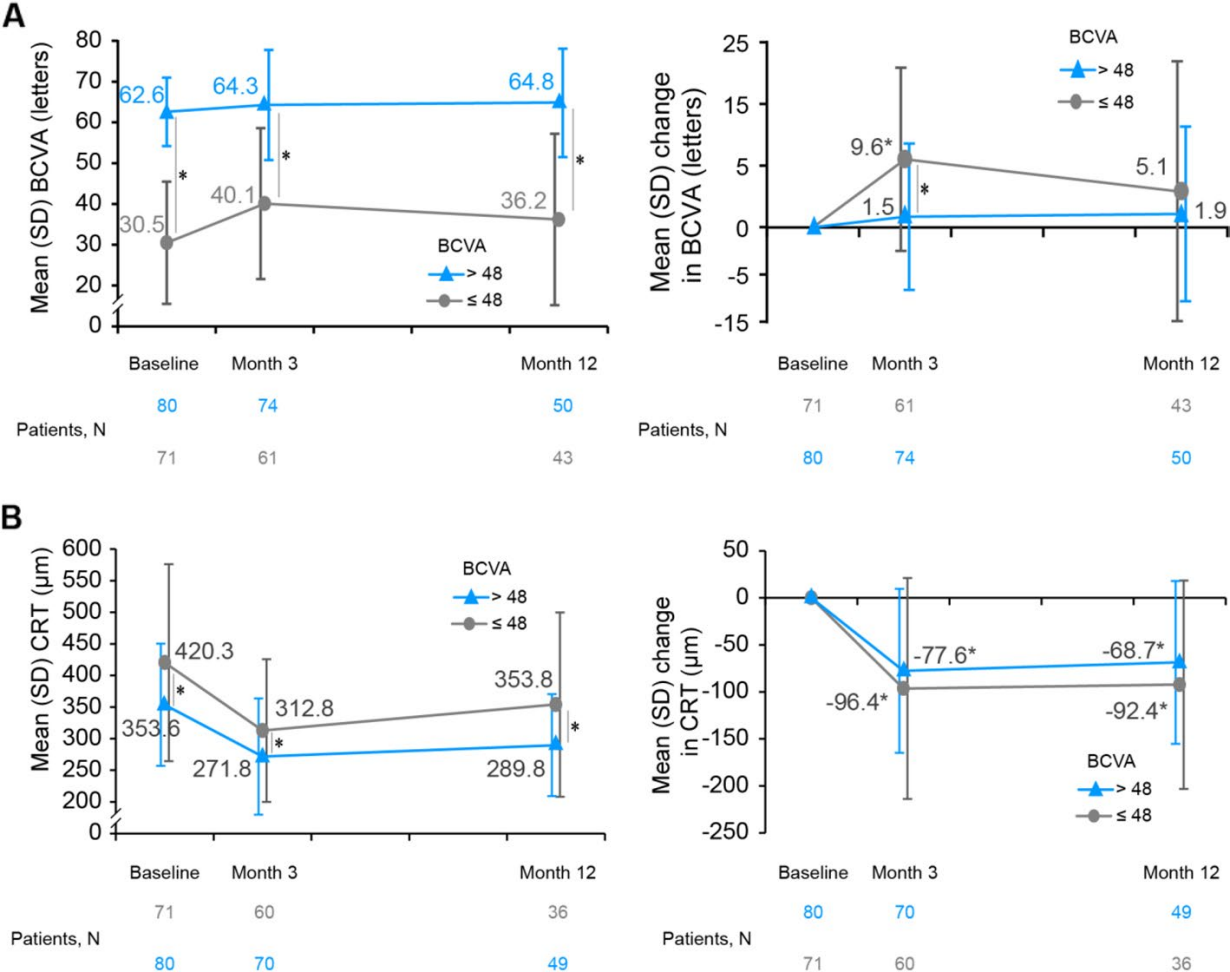
Change from baseline in BCVA and CRT by baseline BCVA (ITT; ≤ 48 letters and > 48 letters). **A** Mean BCVA and mean change in BCVA are presented in adjacent graphs; **B** Mean CRT and mean change in CRT are presented in adjacent graphs. Subgroup with baseline BCVA ≤ 48 letters is represented in grey line; > 48 letter subgroup is represented in blue line. BCVA, best-corrected visual acuity; CRT, central retinal thickness; ITT, intent-to-treat; N, number of patients; VEGF, vascular endothelial growth factors. *Statistical significance. ITT population, all patients who received at least one dose of observational drug (anti-VEGF) and had at least one post-baseline assessment of the study variables.

VA (mean ± SD) improvements at Month 3 were 9.6 ± 12.9 letters in the BCVA ≤ 48 letters group and 1.5 ± 10.3 letters with BCVA > 48 letters group (intergroup *P* < 0.001). The BCVA > 48 letters subgroup maintained significantly better final VA than the BCVA subgroup ≤ 48 letters throughout the study period (intergroup *P* < 0.001 at 3 and 12 months) (Fig. 1A). Furthermore, patients with BCVA ≤ 48 letters had higher baseline CRT than patients with BCVA > 48 letters (420.3 ± 156.0 vs. 353.6 ± 96.7; intergroup *P* = 0.007), which was sustained across the study period (intergroup *P* = 0.023 at Month 3 and 0.020 at Month 12) (Fig. 1B). The number of injections and nAMD duration did not differ based on the baseline BCVA.

#### By baseline CRT

Patients were classified into two subgroups based on severity of CRT at baseline: (1) CRT ≤ 325 µm and (2) CRT > 325 µm. CRT severity at baseline showed no correlation with the age of the patient (Table 1). No significant difference in the number of ranibizumab injections was observed between the two baseline CRT groups (intergroup *P* = 0.463) in the ITT population (Table 1).

**Fig. 2:**
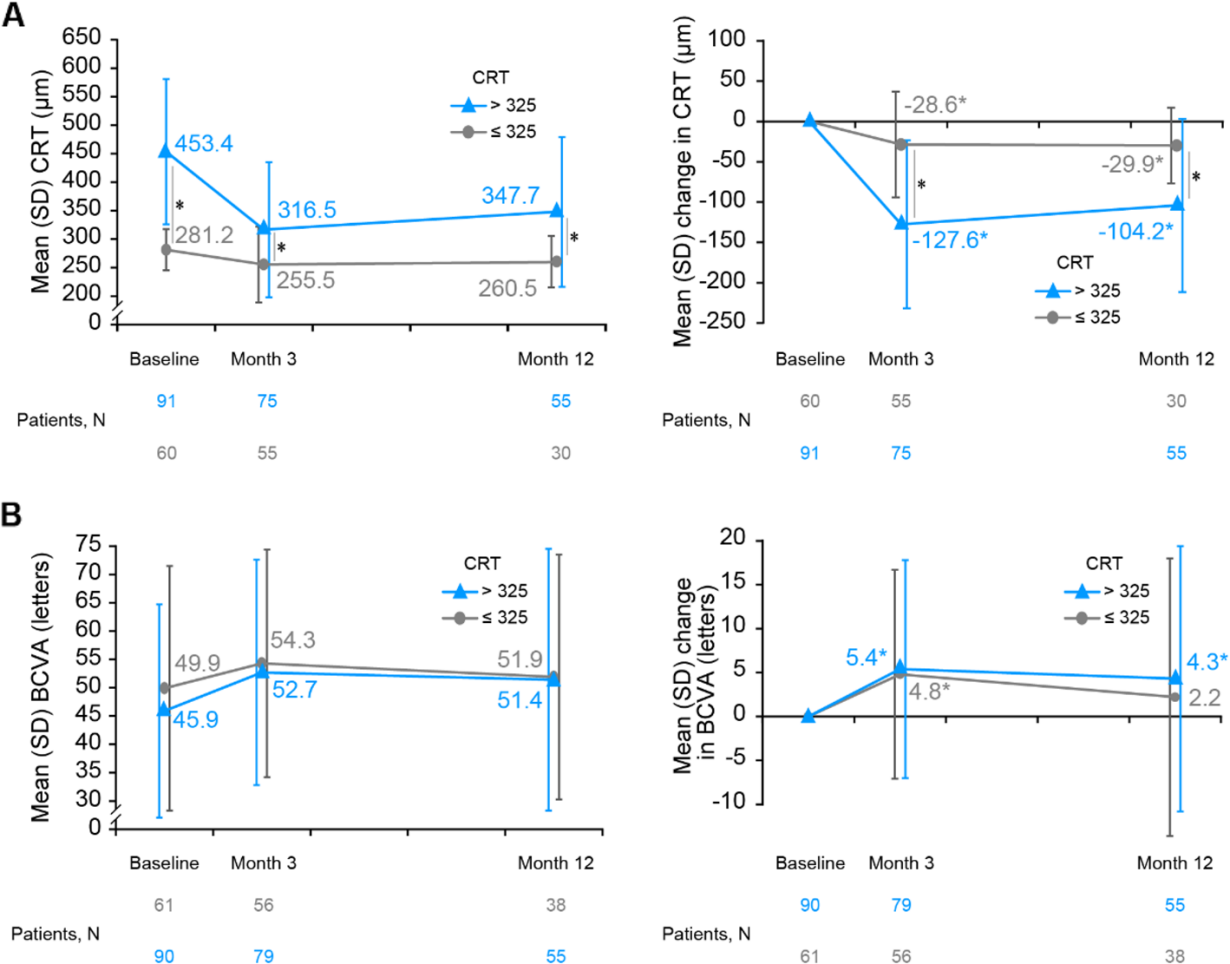
Change from baseline in CRT and BCVA by severity of baseline CRT (ITT; ≤ 325 µm and > 325 µm). **A** Mean CRT and mean change in CRT are presented in adjacent graphs; **B** Mean BCVA and mean change in BCVA are presented in adjacent graphs. Subgroup with baseline CRT ≤ 325 µm is represented in grey line; > 325 µm subgroup is represented in blue line. BCVA, best-corrected visual acuity; CRT, central retinal thickness; ITT, intent-to-treat; N, number of patients; VEGF, vascular endothelial growth factors. *Statistical significance. ITT population, as all patients who received at least one dose of observational drug (anti-VEGF) and had at least one post-baseline assessment of the study variables

Significantly higher reductions of CRT were observed across the study period in the subgroup with baseline CRT > 325 μm (all *P* < 0.001), with reductions of 127.6 ± 104.2 μm at Month 3 and 104.2 ± 107.4 μm at Month 12. In the baseline CRT ≤ 325 μm subgroup, CRT reductions were 28.6 ± 65.6 μm at Month 3 and 29.9 ± 46.8 μm at Month 12 (all *P* < 0.001). The baseline CRT ≤ 325 μm subgroup reached almost a 250 μm threshold in terms of final CRT at both Months 3 and 12 (255.5 ± 66.5 μm and 260.5 ± 45.0 μm, respectively) (Fig. 2A**,** grey line). Changes in BCVA in this subgroup were comparable among patients with different severity of baseline CRT (intergroup *P* = 0.203 and 0.280 at Month 3 and 12, respectively) with 5.4 ± 12.4 letters (95%CI: 2.7 to 8.2 letters) vs. 4.8 ± 11.9 letters (95%CI: 1.6 to 8.0 letters) at Month 3 and 4.3 ± 15.1 letters (95%CI: 0.2 to 8.4 letters) vs. 2.2 ± 15.8 letters (95%CI: –3.0 to 7.4 letters) at Month 12, for baseline CRT > 325 µm and ≤ 325 µm, respectively (Fig. 2B).

### Subgroup analysis by treatment response

Patient subgroups were further analysed to correlate the treatment response at Month 3 in terms of BCVA and CRT improvements. Those receiving three ranibizumab injections in the first 3 months (3M3D population) were considered for this analysis; the regimen of three loading doses was guided by the Taiwan-approved Ranibizumab Label and the NHI nAMD reimbursement criteria during the study period.

#### By treatment response at month 3 (BCVA)

Forty-one patients (38.7%) showed favourable treatment improvement in terms of BCVA gains ≥ 5 letters at Month 3, while 65 patients (61.3%) gained < 5 letters at Month 3. Patient characteristics among the subgroups of treatment response (gains < 5 letters or ≥ 5 letters) are summarised in Table 1 (3M3D population). No significant differences were observed in age, gender, nAMD duration and number of ranibizumab injections between the treatment response subgroups. Baseline BCVA was also comparable between the subgroups; however, baseline CRT was numerically higher in the ≥ 5-letter gain subgroup with borderline significance (*P* = 0.067) (Table 1).

**Fig. 3:**
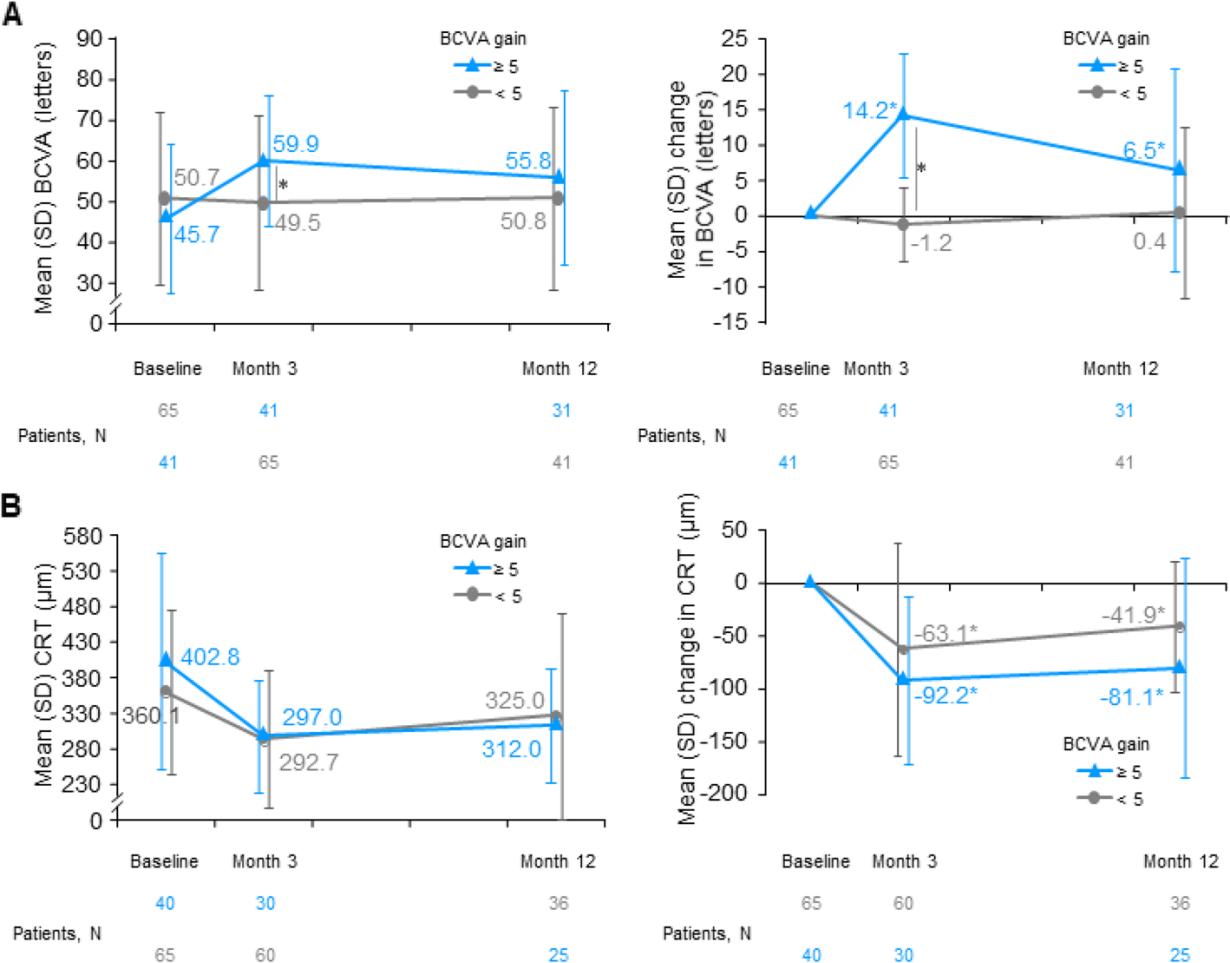
Change from baseline in BCVA and CRT by treatment response in BCVA (3M3D) (< 5-letter and ≥ 5-letter gain). **A** Mean BCVA and mean change in BCVA are presented in adjacent graphs; **B** Mean CRT and mean change in CRT are presented in adjacent graphs. Subgroup with treatment response < 5 letters is shown with grey line; ≥ 5 letter subgroup is shown with blue line. BCVA, best-corrected visual acuity; CRT, central subfield thickness; ITT, intent-to-treat; N, number of patients; VEGF, vascular endothelial growth factors. *Statistical significance. 3M3D population, a subset of ITT population that received all 3 doses of anti-VEGF within 3 months without protocol deviation

BCVA gains were significantly higher for the subgroup with ≥ 5-letter gain at Month 3. These gains at Month 3 (14.2 ± 8.7 letters [95%CI: 11.4 to 16.9 letters]) were followed by a decline in the magnitude of gain at Month 12 (6.5 ± 14.3 letters [95%CI: 1.2 to 11.7 letters]). Nevertheless, the BCVA remained increased at Month 12 compared to baseline (55.8 ± 21.4 vs. 45.7 ± 18.2 letters; Fig. 3A, blue line). A borderline significant difference in BCVA changes remained consistent between the two subgroups at Month 12 (*P* = 0.056).

Similar reductions in CRT were observed in the subgroup with BCVA ≥ 5 letters compared to the < 5 letter subgroup (intergroup *P* = 0.524 and *P* = 0.623 at Month 3 and Month 12, respectively), indicating that treatment response in BCVA might be weakly associated with the changes in CRT. CRT at baseline, although numerically higher in the subgroup with BCVA ≥ 5 letters, was comparable at Month 12 in the < 5 letter subgroup (Fig. 3B). The final CRT at Month 12 was 312 ± 79.8 μm and 325 ± 144.7 μm in the BCVA ≥ 5 letter and < 5 letter subgroup, respectively (*P* = 0.623). In the subgroup gaining < 5 letters, final CRT at Month 12 was maintained reduced from baseline with 41.9 ± 61.2 µm in CRT reduction at Month 12 (*P* < 0.001); the BCVA remained stable (Fig. 3A, grey line), consistent with no numerical increase of > 50 μm in CRT at Month 12 compared with Month 3 (Fig. 3B, grey line).

#### By treatment response at month 3 (CRT)

At Month 3, 65 patients (65.7%) in the 3M3D analysis set responded with a ≥ 50 µm reduction in CRT, while 34 patients (34.3%) exhibited CRT reductions < 50 µm. Patient characteristics among subgroups of treatment response in CRT at Month 3 are summarised in Table 1 (3M3D population). Patients in both the subgroups were comparable in baseline characteristics (age, gender distribution, nAMD duration, baseline BCVA) and number of ranibizumab injections. However, baseline CRT was significantly higher in the CRT reduction ≥ 50 µm subgroup compared to < 50 µm subgroup (*P* < 0.001) (Table 1).

**Fig. 4:**
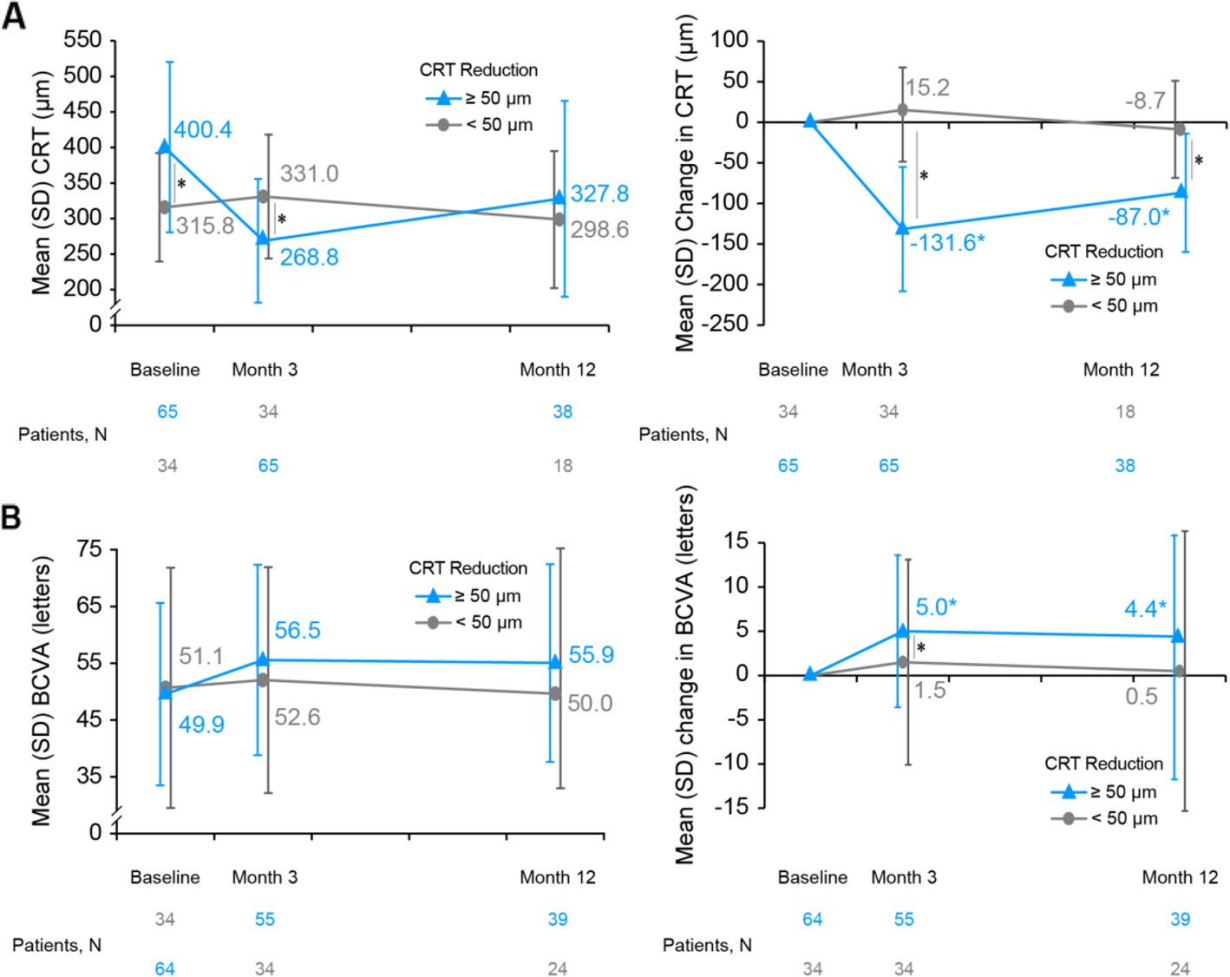
Change from baseline in CRT and BCVA by treatment response in CRT (3M3D; < 50 µm and ≥ 50 µm CRT reduction). **A** Mean CRT and mean change in CRT are presented in adjacent graphs; **B** Mean BCVA and mean change in BCVA are presented in adjacent graphs. Subgroup with < 50 µm CRT reduction is shown in grey line; ≥ 50 µm CRT reduction subgroup is shown in blue line. BCVA, best-corrected visual acuity; CRT, central retinal thickness; ITT, intent-to-treat; N, number of patients; VEGF, vascular endothelial growth factors. *Statistical significance. 3M3D population, a subset of ITT population that received all 3 doses of anti-VEGF within 3 months without protocol deviation

CRT reductions were significantly higher for the CRT ≥ 50 μm subgroup compared with the < 50 µm subgroup at Month 3 (intergroup *P* < 0.001). The significant difference was sustained until Month 12, with a reduction of 87.0 ± 73.0 μm for the patients with ≥ 50 μm reduction and only 8.7 ± 59.8 μm for those with < 50 μm reduction (intergroup *P* < 0.001). Improvement in CRT at Month 3 is associated with the changes in BCVA in terms of significant BCVA improvements over the study period in patients with ≥ 50 μm reduction, with a significant intergroup difference noted at Month 3 (5.0 ± 8.6 letter gains vs. 1.5 ± 11.6 letter gains in the ≥ 50 µm and < 50 μm CRT reduction subgroups, respectively; *P* = 0.005) and a numerically higher BCVA gain at Month 12 (Fig. 4B). The BCVA improvements were maintained at Month 12 in the CRT ≥ 50 μm subgroup with 4.4 ± 11.7 letter gains (95%CI: 0.6 to 8.2 letters; Fig. 4B**,** blue lines), despite a trend of numerical CRT rebound at Month 12 compared with Month 3 (Fig. 4A**,** blue lines).

In patients with CRT < 50 μm reduction, no further CRT reduction and no BCVA gains were observed throughout the study period; both of which maintained stable compared to the baseline (Fig. 4 A and B**,** grey lines).

#### Ophthalmic observations

Colour fundus photography was used to evaluate the findings in the patients’ eyes during the study period. As previously described, around half of the ITT population at baseline recorded haemorrhage (58.0%), subretinal fluid (SRF; 51.3%), while 36.7% patients reported pigment epithelial detachment (PED) and16.0% had scar [15]. For most symptoms except scar, the majority of patients (> 92%) showed improvement or stable conditions over time. Overall, haemorrhage (42% at Month 3 and 46% at Month 12) and SRF (39% at Month 3 and 37% at Month 12) showed significant improvements at Months 3 and 12 on fundus (*P* ≤ 0.001 at all visits; Table S1). In the patient subgroup with baseline CRT > 325 µm, the presence of haemorrhage (65%) and scar (21%) was higher compared with their counterparts. In addition, a significant progression was observed for the natural course in scarring (20.5%) at Month 12 (Table S2).

At baseline, the presence of haemorrhage, SRF, PED or scar was comparable between all the treatment response subgroups (BCVA gain < 5 letters and ≥ 5 letters, CRT reduction < 50 µm and ≥ 50 µm, annual injections of 3 and > 3; Table S2). Significant improvement in haemorrhage (67%) was observed at Month 12 in patients with BCVA gains ≥ 5 letters (*P* = 0.009); Table S3. None of the ophthalmic outcomes worsened in any of the subgroups at Month 12 (Table S3). The SRF improvement rate showed a significant difference in the BCVA gain ≥ 5 letter subgroup compared with the BCVA gain < 5 letter subgroup at Month 3 (53.1% vs. 29.2%, respectively; *P* = 0.036). The SRF improvement rate showed a borderline significant difference in the CRT reduction ≥ 50 μm subgroup compared with the CRT reduction < 50 μm subgroup at Month 3 (42.6% vs. 19.2%, respectively; *P* = 0.078; Table S3).

#### Safety

A total of 94 patients reported 254 AEs, the majority (240 of 254; 94.4%) of which were mild to moderate in severity, with allergic conjunctivitis being the ocular AE with highest incidence (5%); serious AEs were reported in 11.8% of patients [15].

## Discussion

In this subgroup analysis, the RACER study population treated with ranibizumab 0.5 mg was assessed for BCVA and CRT improvements based on patient baseline characteristics (BCVA, CRT) and treatment response (BCVA gains and CRT reduction).

Response to various anti-VEGF therapies is generally dependent on patient characteristics such as age, baseline BCVA, nAMD duration, lesion characteristics and genotype risk alleles. An optimal response to anti-VEGF can be largely defined as a condition where there is resolution of fluid (intraretinal fluid [IRF], subretinal fluid [SRF]), reduction in CRT and/or improvement of ≥ 5 letters in BCVA gain, which is subject to ceiling effect seen with good baseline BCVA [16]. In the current RACER subgroup analysis, we observed that as early as after three monthly initial ranibizumab injections, 38.7% and 65.7% of the patients showed favourable treatment improvements in terms of BCVA gain (≥ 5 letters) and CRT reduction (≥ 50 μm) at Month 3, respectively.

The RACER subgroup result is consistent with the RENOWNED study, a 12-month observational study with ranibizumab use for nAMD in Taiwan [17]. In RENOWNED, 49.3% of the patients gained ≥ 5 letters in BCVA at Month 3 with an overall 71.4% completing the three loading doses during the study period. The CRT reduction, however, was not sustained in the RENOWNED study period due to the limited annual ranibizumab injection number of 3.1 [17], compared to the 4.8 − 5.1 annual injections in the RACER CRT subgroups. The RACER result is also consistent with the AMD-MANAGE study [18], a 24-month observational study with both ranibizumab and aflibercept use for nAMD in Spain. In AMD-MANAGE, a mean number of 5.5 anti-VEGF injections in the initial 12-month study period was administered; 41.9% of the patients gained ≥ 5 letters in BCVA at Month 3 with an overall 84.1% completing the loading dose, and the BCVA gain at Month 3 was only sustained until Month 12 in patients, who have received ≥ 5 annual injections, but not < 5 annual injections [18].

Considering the baseline, the patient subgroup with baseline BCVA > 48 letters in RACER showed significantly less improvement in BCVA compared to the BCVA ≤ 48 letter subgroup throughout the study period. This observation is in alignment with previous studies that reported an inverse relation between baseline BCVA and mean vision gains [19, 20]. In RACER, the subgroup with better baseline BCVA maintained significantly higher absolute BCVA throughout the study period, indicating a ceiling effect despite the lower BCVA gain. Notably, in the BCVA ≤ 48 letter subgroup, the initial BCVA improvements observed decline by the end of the study due to the limited injection number in real life. Similar results were shown by Lo et al. in a 3-year study from Taiwan: the BCVA gain peaked at Month 3 and declined throughout the study period due to limited annual anti-VEGF injection number (4.63) in the first 12 months [21]. In comparison, AMD-MANAGE study showed a sustained BCVA gain that improved continuously in the patient subgroup receiving ≥ 5 annual injections [18].

BCVA gains appeared to be independent of baseline CRT severity (≤ 325 µm and > 325 µm) as the BCVA gains were comparable between the two baseline CRT subgroups (intergroup P = 0.203 and 0.280 at Month 3 and 12, respectively). This observation needs to be interpreted in the light of the previously reported Comparison of AMD Treatment Trials (CATT) sub-analysis that showed greater baseline CRT to be one of the factors associated with reduced visual outcomes [22, 23]. A floor effect may provide an explanation in the patient subgroup with baseline CRT ≤ 325 µm; as the mean change in CRT was comparatively smaller, they attained a final CRT level of < 300 µm, which was sustained throughout the study period. In contrast, a trend of CRT rebound at Month 12 was observed in the subgroup with baseline CRT > 325 µm due to the limited injection number in real life. Similar observations were noted in previous studies where patients with treatment-naive eyes with nAMD may fail to show sustained CRT improvements, unless with continuous treatment [24, 25].

Regarding the treatment response, the 3M3D population comprised those who have received all three doses of anti-VEGF within 3 months; the regimen of three loading doses was guided by the Taiwan-approved Ranibizumab Label and the NHI nAMD reimbursement criteria during the study period. In the subgroup showing a favourable treatment improvement of ≥ 5 letter gains in BCVA at Month 3, the magnitude of gain showed a numerical decline at Month 12 after its peak at Month 3 due to the limited number of injections in the clinical practice setting. The CRT at baseline was not significantly different, and the final CRT at Month 12 was comparable (312.0 ± 79.8 µm and 325.0 ± 144 µm, respectively; intergroup *P* = 0.623) between the subgroups with ≥ 5 letter and < 5 letter gains in BCVA. This indicates that the treatment response in BCVA may be weakly associated with CRT in our findings.

Favourable treatment improvement in terms of CRT reduction (≥ 50 µm) at Month 3 was seen in 65.7% of patients. Despite the trend of numerical CRT rebound at Month 12 in the ≥ 50 µm CRT reduction subgroup, the BCVA gains remained stable with 4.4 ± 11.7 letter gains at Month 12. Notably, in the CRT reduction < 50 μm subgroup at Month 3, the final CRT was still maintained below the level of 300 μm at Month 12. This explained the response of anti-VEGF treatment for nAMD with the floor effect in CRT should consider whether the annual results were maintained at a controlled disease activity level for the final CRT < 300 μm at Month 12.

Observed ophthalmic/anatomical changes revealed that patients with better BCVA response at Month 3 showed greater improvement in SRF at Month 3 inferring that the BCVA gains at Month 3 might be associated with SRF change at Month 3 (P = 0.036). By contrast, the CRT response at Month 12 did not show consistent finding compared with Month 3 and the improvement in SRF was inversely better in the CRT reduction < 50 μm subgroup at Month 12 despite being numerically insignificant. This could be affected by the limited number of cases and/or disease course, such as CRT rebound in the CRT reduction ≥ 50 subgroup.

As per the current Taiwan NHI scheme, nAMD patients eligible for anti-VEGF reimbursement are granted a lifetime of 14 injections per eye [26]; however, the policy does not allow treatment switch [26, 27] and patients are mandated to continue the therapy initially approved regardless of the functional and anatomical response. Therefore, it is not possible to see the outcomes if the suboptimal patient subgroups had been switched in the RACER study period. Benefits of treatment switch were elaborated in a pooled meta-analysis that presented results from 28 studies including 2254 eyes of patients with nAMD. Patients were followed up for 6–24 months after anti-VEGF switch; visual and anatomical outcomes such as BCVA changes/stability and CRT changes were evaluated. Overall, visual function remained stable with no considerable improvements after treatment switch, while significant improvements in anatomical outcomes (CRT reduction) were observed [28]. Earlier studies revealed that at 12 months after treatment switch in a clinical practice setting, patients displayed anatomic improvements in fluid and significant CRT reductions and stabilised the visual acuity that was otherwise trending towards vision loss (prior to switch) along with anatomical improvements [29, 30]. Some studies also reported significant visual and anatomic improvements (CRT reduction) in patients with nAMD at 12 months after anti-VEGF conversion in patients with initial suboptimal response [31, 32]. Considering the available clinical evidence on the benefits of switching, nAMD patients without early favourable treatment response may require long-term anti-VEGF therapy, and the benefit of switch in anti-VEGF agents for better clinical outcomes remains to be investigated in Taiwan with the evolution of the reimbursement policy.

This subgroup analysis considered majority of the disease confounding factors (haemorrhage, SRF, PED, scar) known to impact treatment outcomes with anti-VEGF therapies, providing a comprehensive and qualitative analysis of the treatment response in patients with nAMD, although the IRF data were not documented in RACER due to the design of reporting by investigator discretion instead of implementing a reading centre. The small sample size of the patients analyzed, missing data/loss to follow-up, as in any observational study and no common standard established for the OCT imaging are some of the limitations. In addition, as patients were not randomised and treated as per investigators’ discretion, this could have influenced the treatment response. The subgroup analysis was pre-defined in the Statistical Analysis Plan before the database lock, while the expansion of the reimbursed injection number took place from a previous 3 to 3 + 4 injections for nAMD in Taiwan in August 2014, being in effect during the RACER study period and enabling the subgroup analysis reported herein. The high external validity associated with observational studies serves as a strength as they reflect the current real-world clinical practice scenario. Furthermore, the study does not overestimate the therapeutic efficacy compared to the results of randomised trials.

## Conclusion

In conclusion, the results of our subgroup analysis reiterate the importance of early and intensive treatment in both the optimal and suboptimal response subgroups, for different aspects of sustaining the initial good response or stabilizing the final outcomes to be maintained above the baseline disease control level. The ceiling effect and floor effect were the key drivers for the prognosis in terms of BCVA gains and CRT reductions throughout the study period. While the BCVA improvements cannot be predicted based on baseline CRT severity, the initial improvements in CRT were associated with BCVA gains as early as Month 3. These results demonstrate a potential for treatment guidance, and the unexplored outcomes of switch in anti-VEGF agents for better clinical outcomes remain to be investigated in Taiwan in the suboptimal response groups.

## Supplementary Information

**Additional file 1: Table S1.** Colour fundus photography results at Months 3 and 12 compared to baseline (ITT^a^). **Table S2.** Colour fundus photography findings at baseline, by baseline characteristics (BCVA and CRT) in ITT^a^ population, number of annual injections and treatment response at Month 3 in 3M3D^b^. **Table S3.** Colour fundus photography results by category of ‘improved’ and ‘worsen’ (category of ‘stable’ represents the rest of percentage; therefore, not shown) compared to baseline, by baseline characteristic in ITT,^a^ number of annual injections and treatment response in 3M3D^b^.

## Data Availability

All data provided are anonymised to respect the privacy of patients who have participated in the observational study in line with applicable laws and regulations. The datasets used and/or analysed during this study are available from corresponding author on reasonable request.

## Abbreviations

AE: Adverse event
AMD: Age-related macular degeneration
BCVA: Best-corrected visual acuity
CATT: Comparison of AMD Treatment Trials
CI: Confidence interval
CRT: Central retinal thickness
IRF: Intraretinal fluid
ITT: Intent-to-treat
nAMD: Neovascular age-related macular degeneration
NHI: National Health Insurance
OCT: Optical coherence tomography
RACER: Ranibizumab AMD clinical efficacy in real-world practice
SAE: Serious adverse event
SD: Standard deviation
SRF: Subretinal fluid
VA: Visual acuity
VEGF: Vascular endothelial growth factor
