# Supplementary material for: Baseline characteristics and treatment response predictive of nAMD outcomes with ranibizumab therapy in treatment-naive patients: the RACER subgroup analysis
Source: BMC Ophthalmol. 2023 Jan 27;23:39. doi: 10.1186/s12886-023-02780-0 (PMC9881324; doi:10.1186/s12886-023-02780-0)
Supplement: Supplementary file 1 — Additional file 1: Table S1. Colour fundus photography results at Months 3 and 12 compared to baseline (ITTa). Table S2. Colour fundus photography findings at baseline, by baseline characteristics (BCVA and CRT) in ITTa population, number of annual injections and treatment response at Month 3 in 3M3Db. Table S3. Colour fundus photography results by category of ‘improved’ and ‘worsen’ (category of ‘stable’ represents the rest of percentage; therefore, not shown) compared to baseline, by baseline characteristic in ITT,a number of annual injections and treatment response in 3M3Db. [file 12886_2023_2780_MOESM1_ESM.docx]

**SUPPLEMENTARY INFORMATION**

**Table S1. Colour fundus photography results at Months 3 and 12 compared to baseline (ITT^a^)**

| **ITT (N=152)** | **Improved, n (%)** | **No change, n (%)** | **Worsen, n (%)** | ***P* value** |
| --- | --- | --- | --- | --- |
| **Haemorrhage** |  |  |  |  |
| Month 3 | **46 (42.2)** | 61 (56.0) | 2 (1.8) | <0.001^*^ |
| Month 12 | **31 (46.3)** | 34 (50.7) | 2 (3.0) | <0.001^*^ |
| **SRF** |  |  |  |  |
| Month 3 | **42 (38.5)** | 61 (56.0) | 6 (5.5) | <0.001^*^ |
| Month 12 | **25 (37.3)** | 40 (59.7) | 2 (3.0) | <0.001^*^ |
| **PED** |  |  |  |  |
| Month 3 | 20 (18.3) | 80 (73.4) | 9 (8.3) | 0.061 |
| Month 12 | 8 (11.9) | 51 (76.1) | 8 (11.9) | 1.00 |
| **Scar** |  |  |  |  |
| Month 3 | 4 (3.7) | 92 (84.4) | **13 (11.9)** | 0.049* |
| Month 12 | 1 (1.5) | 54 (80.6) | **12 (17.9)** | 0.003* |

ITT, intent-to-treat; n, number of patients; N, total number of patients; PED, pigment epithelial detachment; SRF, subretinal fluid; VEGF, vascular endothelial growth factor.

^a^ITT population, all patients who received at least one dose of observational drug (anti-VEGF) and had at least one post-baseline assessment of the study variables.

*Statistically significant, significance level=0.05.

**Ophthalmic observations**

***By baseline BCVA***

Colour fundus photography parameters (haemorrhage, SRF, PED, scar) in the ITT population at baseline were comparable between the two baseline BCVA subgroups (Table S2). Changes in haemorrhage, PED and SRF after treatment were comparable between the BCVA subgroups (Table S3).

***By baseline CRT***

In the baseline CRT subgroups, the colour fundus parameters were generally comparable at baseline except for haemorrhage and scar. The proportion of patients with haemorrhage was significantly higher in the CRT >325 μm subgroup compared to the CRT ≤325 μm subgroup (65.2% vs. 47.5%) in the ITT analysis set (Table S2). Changes in all the findings were comparable between patients with different baseline severity of CRT, without any difference in nAMD duration and number of injections. Baseline CRT appears not to be associated with the changes in haemorrhage, PED, SRF and scar (Table S3).

**Table S2. Colour fundus photography findings at baseline, by baseline characteristics (BCVA and CRT) in ITT^a^ population, number of annual injections and treatment response at Month 3 in 3M3D^b^**

| **By baseline characteristics** | | | |
| --- | --- | --- | --- |
| **ITT (N=152)** | **BCVA ≤48 letters, N=72, n (%)** | **BCVA >48 letters,  N=80, n (%)** | ***P* value**^†^ |
| **Haemorrhage** | 45 (64.3) | 42 (52.5) | 0.145 |
| **SRF** | 35 (50.0) | 42 (52.5) | 0.760 |
| **PED** | 21 (30.0) | 34 (42.5) | 0.113 |
| **Scar** | 15 (21.4) | 9 (11.3) | 0.090 |
| **ITT (N=152)** | **CRT ≤325 μm,  N=61, n (%)** | **CRT >325 μm,  N=91, n (%)** | ***P* value**^†^ |
| **Haemorrhage** | 29 (47.5) | 58 (65.2) | 0.032* |
| **SRF** | 26 (42.6) | 51 (57.3) | 0.077 |
| **PED** | 28 (45.9) | 27 (30.3) | 0.052 |
| **Scar** | 6 (9.8) | 18 (20.2) | 0.088 |
| **By number of injections** | | | |
| **3M3D (N=118)** | **3 injections,  N=43, n (%)** | **>3 injections,  N=75, n (%)** | ***P* value**^†^ |
| **Haemorrhage** | 24 (55.8) | 44 (58.7) | 0.763 |
| **SRF** | 17 (39.5) | 42 (56.0) | 0.085 |
| **PED** | 12 (27.9) | 30 (40.0) | 0.187 |
| **Scar** | 6 (14.0) | 10 (13.3) | 0.925 |
| **By treatment response after 3 injections in 3 months** | | | |
|  | **BCVA gain <5 letters, N=65, n (%)** | **BCVA gain ≥5 letters,** **N=41, n (%)** | ***P* value**^†^ |
| **Haemorrhage** | 35 (53.8) | 26 (63.4) | 0.332 |
| **SRF** | 30 (46.2) | 23 (56.1) | 0.319 |
| **PED** | 23 (35.4) | 15 (36.6) | 0.900 |
| **Scar** | 8 (12.3) | 6 (14.6) | 0.730 |
|  | **CRT reduction <50 μm, N=34, n (%)** | **CRT reduction ≥50 μm,** **N=65, n (%)** | ***P* value**^†^ |
| **Haemorrhage** | 19 (55.9) | 36 (55.4) | 0.962 |
| **SRF** | 14 (41.2) | 33 (50.8) | 0.364 |
| **PED** | 14 (41.2) | 22 (33.8) | 0.472 |
| **Scar** | 2 (5.9) | 10 (15.4) | 0.210 |
| BCVA, best-corrected visual acuity; CRT, central retinal thickness; ITT, intent-to-treat; N, total number of patients; n, number of patients; PED, pigment epithelial detachment; SRF, subretinal fluid; VEGF, vascular endothelial growth factors. | | | |

^a^ITT population, all patients who received at least one dose of observational drug (anti-VEGF) and had at least one post-baseline assessment of the study variables.

^b^3M3D population, a subset of ITT population that received all 3 doses of anti-VEGF within 3 months without protocol deviation.

^†^The difference of categorical variables between treatment groups was compared by Chi-square test at a statistical significance level of 0.05. If expected number of any cell was <5, Fisher's exact test was used.

*Statistically significant, significance level=0.05.

**Table S3:** **Colour fundus photography results by category of ‘improved’ and ‘worsen’ (category of ‘stable’ represents the rest of percentage; therefore, not shown) compared to baseline, by baseline characteristic in ITT,^a^ number of annual injections and treatment response in 3M3D^b^**

|  | **ITT (N=152)** | **BCVA ≤48 letters,  n (%)** | | **BCVA >48 letters,  n (%)** | | ***P* value**^†^ |
| --- | --- | --- | --- | --- | --- | --- |
|  |  | **Improved** | **Worsen** | **Improved** | **Worsen** |  |
| **Haemorrhage** | Month 3 | 24 (46.2) | 2 (3.8) | 22 (38.6) | 0 (0.0) | 0.181 |
|  | Month 12 | 18 (54.5) | 1 (3.0) | 13 (38.2) | 1 (2.9) | 0.376 |
| **SRF** | Month 3 | 20 (38.5) | 3 (5.8) | 22 (38.6) | 3 (5.3) | 1.000 |
|  | Month 12 | 13 (39.4) | 2 (6.1) | 12 (35.3) | 0 (0.0) | 0.408 |
| **PED** | Month 3 | 10 (19.2) | 5 (9.6) | 10 (17.5) | 4 (7.0) | 0.852 |
|  | Month 12 | 3 (9.1) | 4 (12.1) | 5 (14.7) | 4 (11.8) | 0.915 |
| **Scar** | Month 3 | 3 (5.8) | 4 (7.7) | 1 (1.8) | 9 (15.8) | 0.294 |
|  | Month 12 | 1 (3.0) | **9 (27.3)** | 0 (0.0) | **3 (8.8)** | 0.040* |
|  | **ITT (N=152)** | **CRT ≤325 μm,  n (%)** | | **CRT >325 μm,  n (%)** | | ***P* value**^†^ |
|  |  | **Improved** | **Worsen** | **Improved** | **Worsen** |  |
| **Haemorrhage** | Month 3 | 16 (36.4) | 1 (2.3) | 30 (46.2) | 1 (1.5) | 0.563 |
|  | Month 12 | 7 (30.4) | 0 (0.0) | 24 (54.5) | 2 (4.5) | 0.066 |
| **SRF** | Month 3 | 15 (34.1) | 3 (6.8) | 27 (41.5) | 3 (4.6) | 0.686 |
|  | Month 12 | 8 (34.8) | 1 (4.3) | 17 (38.6) | 1 (2.3) | 1.000 |
| **PED** | Month 3 | 10 (22.7) | 3 (6.8) | 10 (15.4) | 6 (9.2) | 0.678 |
|  | Month 12 | 3 (13.0) | 3 (13.0) | 5 (11.4) | 5 (11.4) | 1.000 |
| **Scar** | Month 3 | 1 (2.3) | 5 (11.4) | 3 (4.6) | 8 (12.3) | 0.917 |
|  | Month 12 | 0 (0.0) | 3 (13.0) | 1 (2.3) | 9 (20.5) | 0.684 |
|  | **3M3D (N=118)** | **3 injections,  n (%)** | | **>3 injections,  n (%)** | | ***P* value**^†^ |
|  |  | **Improved** | **Worsen** | **Improved** | **Worsen** |  |
| **Haemorrhage** | Month 3 | 14 (48.3) | 1 (3.4) | 23 (37.7) | 1 (1.6) | 0.374 |
|  | Month 12 | 8 (57.1) | 0 (0.0) | 17 (44.7) | 0 (0.0) | 0.536 |
| **SRF** | Month 3 | 10 (34.5) | 1 (3.4) | 25 (41.0) | 5 (8.2) | 0.596 |
|  | Month 12 | 3 (21.4) | 0 (0.0) | 18 (47.4) | 2 (5.3) | 0.146 |
| **PED** | Month 3 | 5 (17.2) | 1 (3.4) | 13 (21.3) | 6 (9.8) | 0.561 |
|  | Month 12 | 0 (0.0) | 2 (14.3) | 7 (18.4) | 4 (10.5) | 0.290 |
| **Scar** | Month 3 | 0 (0.0) | 4 (13.8) | 3 (4.9) | 6 (9.8) | 0.591 |
|  | Month 12 | 0 (0.0) | 2 (14.3) | 1 (2.6) | 9 (23.7) | 0.784 |
|  | **3M3D (N=118)** | **BCVA gain <5 letters,  n (%)** | | **BCVA gain ≥5 letters,  n (%)** | | ***P* value**^†^ |
|  |  | **Improved** | **Worsen** | **Improved** | **Worsen** |  |
| **Haemorrhage** | Month 3 | 19 (39.6) | 0 (0.0) | 14 (43.8) | 2 (6.3) | 0.207 |
|  | Month 12 | **7 (26.9)** | 0 (0.0) | **14 (66.7)** | 0 (0.0) | 0.009* |
| **SRF** | Month 3 | **14 (29.2)** | **5 (10.4)** | **17 (53.1)** | **0 (0.0)** | 0.036* |
|  | Month 12 | 8 (30.8) | 0 (0.0) | 11 (52.4) | 1 (4.8) | 0.100 |
| **PED** | Month 3 | 9 (18.8) | 4 (8.3) | 8 (25.0) | 3 (9.4) | 0.812 |
|  | Month 12 | 4 (15.4) | 2 (7.7) | 3 (14.3) | 4 (19.0) | 0.517 |
| **Scar** | Month 3 | 1 (2.1) | 6 (12.5) | 1 (3.1) | 4 (12.5) | 1.000 |
|  | Month 12 | 0 (0.0) | 4 (15.4) | 0 (0.0) | 7 (33.3) | 0.181 |
|  | **3M3D (N=118)** | **CRT reduction**  **<50 μm, n (%)** | | **CRT reduction**  **≥50 μm, n (%)** | | ***P* value**^†^ |
|  |  | **Improved** | **Worsen** | **Improved** | **Worsen** |  |
| **Haemorrhage** | Month 3 | 6 (23.1) | 1 (3.8) | 26 (48.1) | 1 (1.9) | 0.057 |
|  | Month 12 | 3 (23.1) | 0 (0.0) | 17 (56.7) | 0 (0.0) | 0.054 |
| **SRF** | Month 3 | 5 (19.2) | 3 (11.5) | 23 (42.6) | 3 (5.6) | 0.078 |
|  | Month 12 | 5 (38.5) | 1 (7.7) | 8 (26.7) | 1 (3.3) | 0.424 |
| **PED** | Month 3 | 4 (15.4) | 3 (11.5) | 11 (20.4) | 4 (7.4) | 0.730 |
|  | Month 12 | 1 (7.7) | 1 (7.7) | 5 (16.7) | 5 (16.7) | 0.658 |
| **Scar** | Month 3 | 1 (3.8) | 2 (7.7) | 2 (3.7) | 7 (13.0) | 0.869 |
|  | Month 12 | 0 (0.0) | 3 (23.1) | 1 (3.3) | 6 (20.0) | 1.000 |

BCVA, best-corrected visual acuity; CRT, central retinal thickness; ITT, intent-to-treat; N, total number of patients; n, number of patients; PED, pigment epithelial detachment; SRF, subretinal fluid; VEGF, vascular endothelial growth factor.

^a^ITT population is defined as all patients who received at least one dose of observational drug
(anti-VEGF) and had at least one post-baseline assessment of the study variables.

^b^3M3D population is defined as a subset of ITT population that received all three doses of
anti-VEGF within 3 months without protocol deviation.

^†^The differences in continuous variables between treatment groups were compared by independent *t* test at a statistical significance level of 0.05. If the data had not been well modelled by a normal distribution, the Mann-Whitney *U* test would be used.

*Statistically significant, significance level=0.05.
